# Prevalence of Comorbid Postpartum Depression and Anxiety in Birthing Parents Seeking Treatment for Postpartum Depression: Prévalence de la présence comorbide de la dépression et de l’anxiété post-partum chez les parents ayant accouché qui ont consulté en raison de la dépression post-partum

**DOI:** 10.1177/07067437261462698

**Published:** 2026-07-01

**Authors:** Karley V. George, Calan D. Savoy, Haley Layton, Peter J. Bieling, Ryan J. Van Lieshout

**Affiliations:** 1 Neuroscience Graduate Program, 3710 McMaster University, Hamilton, Ontario, Canada; 2 Department of Psychiatry and Behavioural Neurosciences, 3710 McMaster University, Hamilton, Ontario, Canada; 3 Health Research Methodology Graduate Program, 3710 McMaster University, Hamilton, Ontario, Canada

**Keywords:** postpartum depression, postpartum anxiety, comorbidity, prevalence, perinatal mental health, cognitive behavioural therapy, treatment-seeking

## Abstract

**Objective:**

To estimate the prevalence of comorbid postpartum depression (PPD) and postpartum anxiety (PPA) in birthing parents seeking treatment for PPD in Ontario, Canada and who were enrolled in 1 of 9 randomized controlled trials (RCTs) of cognitive behavioural therapies (CBT) for PPD.

**Methods:**

Secondary analysis of 9 pooled RCTs containing baseline data from 1920 birthing parents and conducted between 2017 and 2025. All participants were living in Ontario, Canada with Edinburgh Postnatal Depression Scale (EPDS) Scores ≥10 and infants <12 months old. Comorbid anxiety was assessed using the Generalized Anxiety Disorder-7 (GAD-7) scale, the Penn State Worry Questionnaire (PSWQ) and/or the Mini International Neuropsychiatric Interview (MINI).

**Results:**

Sixty-six percent of participants with PPD (EPDS scores ≥10) had moderate to severe anxiety (GAD-7 scale score ≥10; 95% confidence interval (CI) 63–68%, 7 studies, *n* = 1654), and 69% with PPD met the cutoff for probable GAD (PSWQ scale score ≥61; 95% CI [63–74%], 2 studies, *n* = 254). Seventy percent of participants with MINI-defined current major depressive disorder (MDD) met *Diagnostic and Statistical Manual of Mental Disorders—4th Edition*-based criteria for any anxiety disorder (95% CI [64–76%], 6 studies, *n* *=* 448). Nearly 60% of participants with current MDD met criteria for GAD on the MINI (95% CI [52–61%], *n* = 448).

**Conclusions:**

More than two-thirds of treatment-seeking individuals with PPD have clinically significant anxiety. Those with PPD, their families, and healthcare professionals should be aware of the high prevalence of anxiety in these individuals so that treatment plans can be optimized to best meet their needs.

**Trial Registration:**

ClinicalTrials.gov (https://clinicaltrials.gov/): NCT03039530, NCT03654261, NCT03285139, NCT04485000, NCT04928742, NCT04934488, NCT04913584, NCT05314361 and NCT05044455.

## Résumé

**Objectif:**

Estimer la prévalence de la présence comorbide de la dépression post-partum (DPP) et de l’anxiété post-partum (APP) chez les parents ayant accouché qui ont consulté en raison de la DPP en Ontario, au Canada, et qui ont été inscrits à l’un des neuf essais contrôlés randomisés (ECR) portant sur les thérapies cognitivo-comportementales (TCC) pour la DPP.

**Méthodes:**

Analyse secondaire de neuf ECR groupés comprenant des données initiales provenant de 1 920 parents ayant accouché et réalisés entre 2017 et 2025. Tous les participants vivaient en Ontario, au Canada, et présentaient un score ≥ 10 sur l’échelle d’évaluation de la dépression postnatale d’Édimbourg (EPDS) et étaient parents d’un nourrisson âgé de moins de 12 mois. L’anxiété comorbide a été évaluée à l’aide des échelles « *Generalized Anxiety Disorder*-7 » (GAD-7), *Penn State Worry Questionnaire* (PSWQ) et/ou *Mini International Neuropsychiatric Interview* (MINI).

**Résultats:**

Soixante-six pour cent des participants atteints de DPP (scores EPDS ≥ 10) présentaient une anxiété modérée à grave (score GAD-7 ≥ 10; intervalle de confiance [IC] à 95%: 63 à 68%, sept études, *n* = 1 654), et 69% des participants atteints de DPP avaient atteint le seuil de TAG probable (score PSWQ ≥ 61; IC à 95%: 63 à 74%, deux études, *n* = 254). Soixante-dix pour cent des participants atteints d’un trouble dépressif majeur (TDM) actuel selon la définition du MINI satisfaisaient aux critères de la 4^e^ édition du Manuel diagnostique et statistique des troubles mentaux (DSM) pour un trouble anxieux quelconque (IC à 95%: 64 à 76%; six études; *n* *=* 448). Près de 60% des participants présentant actuellement un TDM satisfaisaient aux critères relatifs au TAG de l’échelle MINI (IC à 95%: 52 à 61%; *n* = 448).

**Conclusions:**

Plus des deux tiers des personnes atteintes de DPP qui consultent présentent une anxiété cliniquement significative. Les personnes atteintes de DPP, leurs familles et les professionnels de la santé doivent prendre conscience de la forte prévalence de l’anxiété chez ces personnes afin que les plans de traitement puissent être optimisés pour mieux répondre à leurs besoins.

## Background

Postpartum mental disorders are common and are associated with significant personal burden and healthcare costs. Postpartum depression (PPD) affects about 17% of individuals who have given birth worldwide (hereafter referred to as birthing parents),^1^ and is associated with lifetime costs of up to $140,000 (CAD) per case when symptoms and costs during pregnancy are also considered.^2^ Postpartum anxiety (PPA) affects approximately 10% of birthing parents^3^ and costs up to $65,000 (CAD) per case.^2^ These postpartum mental health problems are associated with an increased risk of poorer parenting quality,^4^ reduced attachment between caregiver and baby,^4^ changes in early infant development,^4^ and poorer perceived partner relationship quality.^5^ While the *Diagnostic and Statistical Manual of Mental Disorders—5^th^ Edition (DSM-5)* now contains a peripartum onset specifier for major depressive disorder (MDD) (i.e., onset during pregnancy or the first 4 weeks postpartum),^6^ it does not yet contain perinatal-specific criteria for any other disorder. As a result, the *DSM-5* requires that PPA be labelled as generalized anxiety disorder, panic disorder (PD), agoraphobia, and/or social anxiety disorder (SAD), or as a problem previously classified as an anxiety disorder (e.g., obsessive-compulsive disorder, posttraumatic stress disorder.) PPD and PPA are also frequently defined using self-report questionnaires that quantify symptom severity and/or have cutoffs for probable disorders (e.g., the Generalized Anxiety Disorder 7-item scale (GAD-7)^7^ or the Edinburgh Postnatal Depression Scale (EPDS)).^8,7,9,10^

Regardless of how they are defined, depression and anxiety are thought to be highly comorbid during pregnancy and the postpartum period. This comorbidity is important because birthing parents with both problems may have greater symptom severity than those with either alone,^11,12^ and report more infant temperament challenges and poorer social support.^11^ Comorbid anxiety and depression in general population samples is also associated with a longer time to clinical recovery,^13^ lower rates of remission,^14^ and higher healthcare costs.^13^

Estimates of rates of PPA in those who have PPD have varied widely, and range from 20% to 71%.^12,15–29^ However, this may be contributed to by substantial variation in the measures and thresholds used to define PPA,^22,24^ inclusion of relatively small PPD subsamples,^16,17,20,21,24–26,28^ and greater representation of high-risk individuals (e.g., low income or NICU populations).^21–23,26,28^ Most importantly, few studies have estimated the prevalence of PPA in those with PPD who were actively seeking treatment for their depression. In contrast, most prior studies focused on routine screening,^15,16,22,23^ risk factors,^12,19,21,28^ screening tool optimization,^16,17^ or symptom trajectories.,^18,24^ and therefore included largely non-treatment-seeking samples. However, this treatment-seeking group is important because they come to healthcare providers looking for help and may be more likely to need and undertake treatment.

Examining the prevalence of comorbid PPA in those seeking treatment for PPD is also important for several other reasons. First, in the general population, anxiety often precedes depression,^30^ and the time to first depression treatment is shown to be significantly accelerated by comorbid conditions including generalized anxiety and PD.^31^ Second, since comorbidity is associated with greater symptom severity,^32^ and symptom severity is a facilitator of treatment-seeking,^33^ it is possible that treatment-seeking individuals have higher rates of comorbidity. Furthermore, there is a lack of awareness of PPA compared to PPD^34^ despite substantial overlap of symptoms,^35^ which may cause birthing parents to seek (or receive) treatment for depression rather than anxiety even when anxiety might be their primary concern. Finally, a substantial portion of the observed comorbidity between anxiety and depression is thought to reflect shared variance attributable to dispositional negative affectivity or neuroticism, a stable temperament characterized by heightened emotional reactivity to stress.^36^ Not considering this overlap and shared risk factors between postpartum depression and anxiety^11,19,37^ ignores important potential treatment targets.

Therefore, our focus on a PPD treatment-seeking population may reveal previously unrecognized cases of anxiety that are not adequately targeted in treatment and highlight the need for more comprehensive screening and treatment planning.

Given this background, the objective of the present study was to estimate the prevalence of comorbid PPA in individuals who were seeking treatment for PPD using self-report questionnaires and diagnostic interviews. Participants were enrolled in 1 of 9 randomized controlled trials (RCTs) of cognitive behavioural therapy (CBT)-based treatments for PPD in Ontario, Canada.

## Methods

### Participants

Secondary analysis of 9 pooled RCTs containing baseline data from 1920 de-identified birthing parents and conducted between 2017 and 2025. They entered their trial after having been referred to by healthcare professionals or community organizations or self-referring. Each trial used identical inclusion criteria: birthing parents (ages ≥18 and above) who had an infant less than 12 months old, lived in Ontario, Canada, and had an EPDS Scores ≥10. Participants with bipolar, psychotic, borderline personality, and/or current substance use disorders (assessed using the Mini International Neuropsychiatric Interview (MINI)) were excluded in all but 3 studies.^38–40^

The objectives of this study were to calculate in postpartum participants with an EPDS ≥ 10: (1) the prevalence of symptoms consistent with moderate to severe anxiety (GAD-7 scale score ≥10) symptomology; (2) the prevalence of symptoms meeting the threshold for probable generalized anxiety disorder (Penn State Worry Questionnaire (PSWQ) scale score ≥61); (3) the prevalence of any anxiety disorder using the MINI in participants with current MDD (MINI); (4) and the prevalence of generalized anxiety disorder using the MINI in participants with current MDD (MINI). Estimates of comorbidity in this study utilized measures of PPD and PPA taken at study baseline, that is before the application of any CBT-based intervention.

The interventions offered were either a 9-week group CBT intervention^41–44^ or a 1-day group CBT-based workshop.^38–40,45^ Treatment providers were recovered peers with lived PPD experience,^38,41,42,46^ public health nurses,^43–45^ or specialized mental healthcare professionals.^39,40^ Three of the studies delivered interventions in-person,^40,41,43^ while the remaining were delivered online. One study was identified as a pilot RCT,^45^ while the remaining were full-scale RCTs. Four of the 9 trials used a waitlist control design,^38,40–42^ while the others compared CBT plus treatment as usual to treatment as usual alone.

### Measures

#### Postpartum depression

The EPDS is a 10-item self-report measure of frequency and severity of PPD over the past 7 days and is the most widely used screening tool in perinatal depression care.^8,47^ Total scores range from 0 to 30, where higher scores indicate higher levels of depression.^47^ A score ≥10 is recommended in research to define possible PPD and to keep false negative rates below 10%.^8^ The Cronbach's alpha for the EPDS in our pooled sample was 0.81 with individual study values ranging from 0.75 to 0.83 (Supplemental Table S2).

The MINI is a structured diagnostic interview with validation based on the DSM-IV or 5 and the International Classification of Diseases, 10th revision (ICD-10).^35^ Current MDD criteria were used to define PPD on the MINI.

#### Postpartum anxiety

The GAD-7-item scale is a self-report measure, with a score range of 0 to 21 where higher scores represent more severe generalized anxiety.^7^ Participants report how often they have been bothered by anxiety symptoms (e.g., anxiousness, uncontrollable worry, restlessness, etc.) over the past 2 weeks. In this study, PPA via the GAD-7 was defined as a score ≥10 (i.e., moderate to severe), which has a good sensitivity and negative predictive value (NPV) for GAD in both general population primary care clinics (89% sensitivity, 99% NPV)^7^ and perinatal birthing parents referred for psychiatric consultation (76% sensitivity, 82.5% NPV).^27^ The GAD-7 can also be analyzed using different severity levels including mild (5–9), moderate (10–14) and severe (≥15) cutoffs.^7^ These categories have construct validity in the general population for the number of disability days, number of physician visits, and symptom-related difficulties.^7^ The pooled Cronbach alpha value for the GAD-7 for the 9 studies was 0.85 with individual study values ranging from 0.80 to 0.87 (Supplemental Table S2).

The PSWQ is a 16-item self-report scale (*α* = 0.86)^48^ with a score range from 16 to 80 where higher scores indicate higher worry levels. In this study, PPA on the PSWQ was defined as a score ≥61 which has a sensitivity of 79.1% and specificity of 65.0% to identify probable cases of GAD in the postpartum period.^49^ For the 2 studies that used the PSWQ in our analyses, the individual study values were 0.86 ^43^ and 0.90,^44^ with a pooled Cronbach alpha value of 0.88 (Supplemental Table S2).

The MINI has good specificity for detecting anxiety-based disorders including current PD (0.93), current agoraphobia (AG) (0.88), current SAD (0.86), and current GAD (0.86),^50^ the disorders we used to define our construct of “any anxiety disorder.” In this study, having “any DSM-defined PPA disorder in those with MDD” was defined as the proportion of participants with MDD on the MINI who met criteria for one or more of the above anxiety disorders. This definition was chosen to exclude OCD and PTSD given its similarity to existing studies^17,20^ and as it is the most recent classification of anxiety disorders in the *DSM-5*. (The *DSM-IV* MINI manual was used for our trials with minimal changes between the fourth and fifth version). For comparability to our GAD-7 and PSWQ scale-based definitions, we also examined rates of comorbid current GAD alone in those with MDD.

### Statistical Analysis

Descriptive statistics were calculated using IBM SPSS Statistics (Version 31).^51^ A secondary-analysis was done using baseline (pre-treatment) data from 9 pooled RCTs. Seven of these studies used the GAD-7,^38–42,45,46^ 2 used the PSWQ,^43,44^ and 6 used the MINI.^41–46^ As a result, 1920 birthing parents who had completed baseline measurements between 2017 and 2025, (which included one or more of the above anxiety measures), were included. The R 4.5.2. “meta” package (version 8.2-1) was used,^52^ which included the metaprop function, a random effects model, inverse variance method, restricted maximum-likelihood estimator, and logit transformation. A meta-regression was run in R for pandemic status (recruitment taking place pre, during, or post-covid), and income. To assess study heterogeneity (*I*^2^), values of 25, 50 and 75 were considered low, moderate, and high, respectively.^53^ A 95% confidence interval was reported for each study and a *P*-value < .05 was considered statistically significant. A sensitivity analysis was performed using an EPDS cutoff of 13.^47^

## Results

### Baseline Characteristics of Participants

The baseline characteristics of participants enrolled in the 9 RCTs are summarized in Table 1. The mean age of birthing parents across all trials was 31.98 (*SD* = 4.31) years, and the mean age of their infants was 5.46 months (*SD* = 3.45) (Table 1). Approximately two-thirds of the total sample was White, and most were married or common law. While the total mean years of education was 15.97 (*SD* = 2.06), the mean household incomes varied across studies from $69,347.85 to $120,609.06 (Canadian Dollars, *SD* = 45,451) (Table 1). The mean EPDS score across all study participants was 15.80 (*SD* = 4.38) and the mean GAD-7 score was 12.08 (*SD* = 4.97)

**Table 1.** Baseline Characteristics of Participants.

Study	Measurement Tools	Infant Age, Months, *M* (*SD*)	Mother Age, Years, *M* (*SD*)	Education, Years, *M* (*SD*)	Household Income, $CAD, *M* (*SD*)	Marital/Common Law Status (%)	White Ethnicity (%)	EPDS Total Score (T1), *M* (*SD*)
Amani et al., 2021	A, B, C	5.30 (3.83)	31.11 (4.97)	14.50 (1.44)	69,347.85 (23,166.48)	92.3	94.2	16.40 (3.84)
Van Lieshout et al., 2021	A, C	5.28 (3.39)	31.81 (4.48)	16.77 (2.35)	98,231.38 (50,491.75)	93.9	72.2	16.14 (4.29)
Van Lieshout et al., 2022	A, B, D	5.51 (3.22)	30.87 (4.79)	17.94 (3.38)	79,650.00 (42,883.77)	93.0	91.9	15.90 (4.12)
Huh et al., 2023	A, B, D	6.12 (4.58)	31.56 (4.75)	14.82 (1.78)	92,442.75 (43, 257.35)	89.3	79.4	14.72 (4.58)
Van Lieshout et al., 2023	A, C	5.40 (3.60)	32.30 (4.76)	15.43 (2.14)	81,054.30 (18,727.85)	91.7	64.4	15.45 (4.54)
Babiy et al., 2024	A, C	5.96 (3.16)	32.35 (4.29)	16.14 (2.09)	120,609.06 (62,496.46)	94.1	65.2	16.20 (4.56)
Merza et al., 2024	A, B, C	4.63 (3.07)	31.60 (4.70)	15.18 (1.45)	96,266.87 (42,128.01)	93.2	79.1	16.00 (4.60)
Layton et al., 2025	A, B, C	5.03 (3.22)	31.39 (3.78)	15.71 (1.62)	109,909.09 (45,429.81)	91.8	58.2	16.12 (4.28)
Mansoor et al. (manuscript)	A, B, C	5.53 (3.26)	32.95 (4.65)	15.87 (2.05)	115,376.34 (50,680.43)	94.1	64.5	15.19 (3.93)
Total (*n* = 1920)		5.46 (3.45)	31.98 (4.31)	15.97 (2.06)	98,839.381 (45,450.61)	93.6	69.3	15.80 (4.38)

Abbreviations: A, Edinburgh Postnatal Depression Scale; B, MINI International Neuropsychiatric Interview; C, Generalized Anxiety Disorder 7-item Scale; D, Penn State Worry Questionnaire; *M*, mean; *SD*, standard deviation; CAD, Canadian dollar; T1, pre-intervention baseline data.

### Prevalence of Moderate to Severe PPA (GAD-7 Scores≥10)

**Figure 1.**
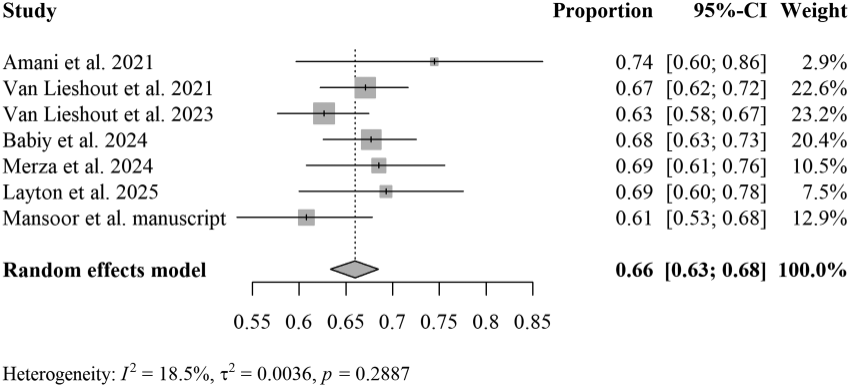
Prevalence of moderate to severe generalized anxiety (≥10 on GAD-7) in postpartum participants with an EPDS ≥ 10.

Abbreviations: GAD-7, generalized anxiety disorder 7-item scale; EPDS, Edinburgh Postnatal Depression Scale.

Approximately two-thirds (66%) of participants with PPD (EPDS≥10) had moderate or severe postpartum GAD-7 score ≥10 based on the GAD-7 (95% confidence interval (CI) 63% to 68%, 7 studies, *n* = 1654) (Figure 1). The rates of anxiety (GAD-7) categorized by mild, moderate and severe categories are shown in Supplemental Table S1. There was low to moderate heterogeneity in the data across all cutoffs (*I*^2^: 0–31.6%) (Figure 1 and Supplemental Table S1), and there were no statistically significant differences in estimates between studies (Figure 1 and Supplemental Table S1).

### Prevalence of Probable Generalized Anxiety Disorder (PSWQ≥61)

**Figure 2.**
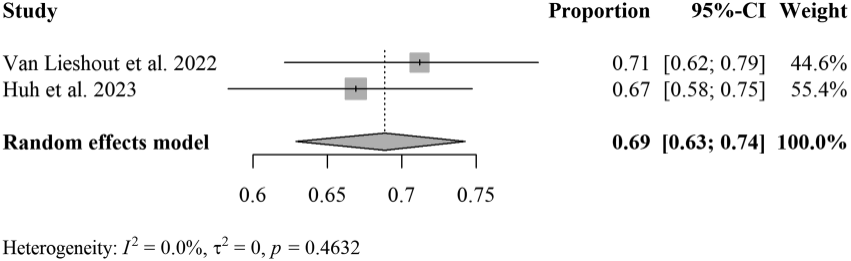
Prevalence of probable GAD (≥61 on the PSWQ) in postpartum participants with an EPDS ≥ 10.

Abbreviations: GAD, generalized anxiety disorder; PSWQ, Penn State Worry Questionnaire; EPDS, Edinburgh Postnatal Depression Scale.

Sixty-nine percent of participants with PPD (EPDS≥10) had probable PPA based on the PSWQ (scale score ≥61) (95% CI [63–74%], 2 studies, *n* = 254) (Figure 2). There was low heterogeneity in the data (*I*^2^: 0%) and there were no statistically significant differences between studies (*P* = .463) (Figure 2).

#### Prevalence of anxiety disorders on the MINI in participants with current MDD

**Figure 3.**
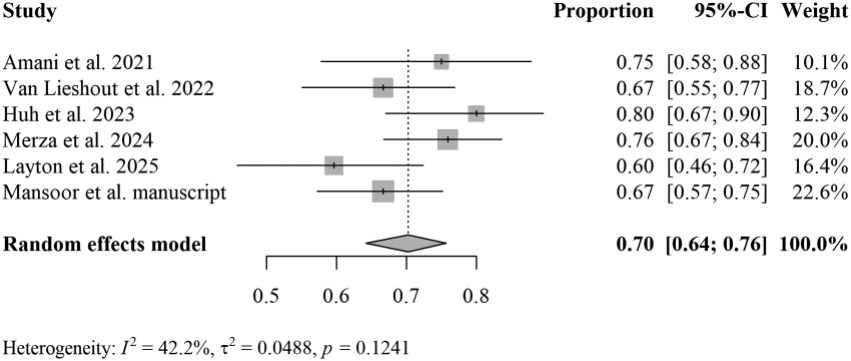
Prevalence of any anxiety disorder (MINI) in participants with current major depressive disorder (MINI).

Abbreviations: any anxiety disorder = one or more of panic disorder, agoraphobia, social anxiety disorder or generalized anxiety disorder. MINI, Mini International Neuropsychiatric Interview.

Approximately 70% of participants with current MDD (MINI) met *DSM*-based criteria for any anxiety disorder (e.g., GAD, SAD, AG, PD) (95% CI [64–76%], 6 studies, *n* *=* 448) (Figure 3).

**Figure 4.**
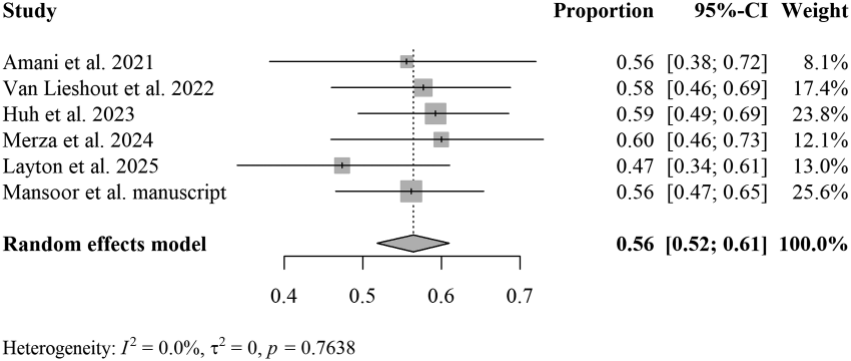
Prevalence of generalized anxiety disorder (MINI) in participants with current major depressive disorder (MINI).

About 56% of those with MDD met criteria for GAD (95% CI [52–61%], *n* = 448) (Figure 4). There was low to moderate heterogeneity and no statistically significant differences in heterogeneity for any anxiety disorder or for GAD, in those with MDD.

Abbreviation:#MINI, Mini International Neuropsychiatric Interview.

#### Meta-regression analysis

No statistically significant differences were found between studies for income or COVID phase for all analyses except the prevalence of any anxiety disorder (MINI) in those with MDD (MINI). The joint test of moderators for this analysis also did not reach significance (*P* = .052), but within this model, post-pandemic studies showed significantly lower prevalence of any anxiety disorder (MINI) in those with MDD than studies that were conducted during the pandemic (*β* = −1.99, *P* = .031).

#### Sensitivity analyses

A sensitivity analysis using only participants with an EPDS≥13 revealed: (1) 77% of participants with an EPDS≥13 had moderate to severe anxiety symptomology on the GAD-7 (95% CI [75–79], 7 studies, *n* = 1294), (2) 80% of participants with an EPDS ≥ 13 had probable GAD on the PSWQ (scale score ≥61) (95% CI [73–85%], 2 studies, *n* = 185), (3) 73% of those with MDD (MINI) met criteria for any anxiety disorder (MINI) 95% CI [68–78%], 6 studies, *n* = 395, and 4. Fifty-nine percent of those with MDD (MINI) met criteria for GAD (MINI) 95% CI [55–64%], 6 studies, *n* = 395).

## Discussion

This work examining the prevalence of PPA in birthing parents with PPD and seeking treatment through both self-report questionnaires and structured diagnostic interviews. These results suggest that at least two-thirds of these individuals have clinically significant PPA based on self-report questionnaires, and of those who met criteria for current MDD, approximately 70% who were seeking treatment met criteria for any anxiety disorder. Importantly, our study specifically targets a highly defined, treatment-seeking clinical trial population with diagnosed PPD using a uniform, multitrial framework, which maximizes internal validity for clinicians managing treatment-seeking patients. Post-pandemic studies showed significantly lower prevalence of any anxiety disorder (MINI) among individuals with MDD than studies conducted during the pandemic, a pattern consistent with prior systematic review on findings that comorbidity proportions were higher during the COVID-19 period.^9^

The prevalence of PPA symptomology among those with PPD in previous studies has ranged from 20% to 71% using self-report measures.^12,18,19,21–24,26,28^ Despite our hypothesis that treatment-seeking birthing parents might experience higher rates of this comorbidity, our GAD-7 and PSWQ-based results are consistent with previous self-reports in non-treatment-seeking samples, albeit at the higher end of estimates. For example, Farr and colleagues (2014), Nakić Radoš et al. (2018) and Tham et al. (2016), all have self-report prevalence rates within 5% of ours (64–69% of birthing parents with PPD from these studies had PPA).^12,18,28^ However, it is possible that participants in these studies were inherently treatment-seeking as they were often recruited from healthcare settings and signed up for studies whose objective in most cases may have been to screen for PPD and PPA.

The prevalence of any anxiety disorder among those with MDD in previous postpartum studies using structured diagnostic interviews has ranged from 34% to 63%. The discrepancy between our results and those of prior studies with lower estimates may be related to the treatment-seeking nature of our sample. However, several of these studies^17,20,25^ were recruited nearly 2 decades ago and recent reports suggest that the prevalence of PPD and PPA in the United States may have doubled during the 2010s.^54^ It is also possible that our use of a different structured diagnostic interview (MINI) contributed to these differences. Two interview-based studies interviews that had similar findings to ours (within 15%) were by Wisner et al. (2015) and Grant et al. (2008).^15,16^ Despite having smaller depression sample sizes, these studies may be more comparable, as one used an EPDS ≥ 10 prior to administering the SCID (similar to our study), and another used the same MINI structured interview version as our studies did (MINI DSM-4th edition).

Among participants with PPD in our analysis, the proportion of those who experienced anxiety was greater when a more sensitive EPDS cutoff of 13 was used rather than 10. This finding may be consistent with existing literature that suggests comorbid depression and anxiety is associated with greater symptom severity than having depression alone.^32^ Regardless, using either cutoff, it is evident based on our findings that birthing parents seeking treatment for PPD have high levels of anxiety. Gender roles and expectations may influence postpartum mental health through caregiving burden, relationship strain, identity changes, and barriers to help-seeking, and could also be valuable factors to consider in future research when interpreting comorbidity prevalence rates. Qualitative interviews with Canadian birthing parents with PPA suggest that they may experience an overwhelming burden of responsibility, gender imbalance or unequal division of tasks in parenthood, and the pressure to conform to societal expectations.^55^ There is also limited research on postpartum mental health in non-birthing parents that could be addressed, with PPD in this group only recently beginning to receive growing attention in the field.^56^

A recent longitudinal study in China by Shen et al. (2024) on comorbidity prevalence suggested a U-shape pattern, with PPD and anxiety symptoms highest in the first trimester, decreasing in the second trimester and increasing again during the third trimester and post-birth.^57^ Notably, very few of their comorbid birthing parents sought treatment during pregnancy or postpartum. Placing these results in context with our own findings raises an important question: do our high comorbidity rates reflect a group with particularly severe symptoms who are more likely to seek treatment, or might similar levels of comorbidity also be present in non-treatment-seeking depressed birthing parents who face barriers to detection and to care? More research on barriers and facilitators to treatment-seeking and uptake for comorbid PPD and anxiety treatment could have wider implications to improve access to care within a resource-constrained public healthcare system.

This study is not without limitations, however. First, our sample was predominantly White, educated, and partnered, and had access to universal healthcare in Ontario, Canada. While these factors can limit the generalizability of our findings, this work suggests that high rates of comorbidity exist even in birthing parents who may be advantaged and have access to healthcare. Second, all studies were completed in the province of Ontario in Canada. Although our sample size was large and utilized participants from across this diverse province of 16.2 million people, the distribution of prevalence rates of co-morbid PPA and depression has shown to vary across countries.^9^ The same levels of comorbidity may not be present in other places in the world. Our exclusion criteria also limited the number of participants with bipolar, psychotic, and borderline personality disorders in most trials. Therefore, our conclusions must be interpreted cautiously for patients who have complex, severe presentations. Regarding the measurements we used, a portion of our findings were derived from self-report questionnaires, and while we also had structured interview data, we did not have access to formal clinician diagnoses. The cutoff of 10 used for the EPDS technically defines possible depression.^47^ However, the fact that our MINI-based definition of MDD yielded similar results, suggests that this cutoff was reasonable. We also used 2 self-report measures of generalized anxiety to measure PPA (GAD-7 and PSWQ). However, these had results within 3% of each other. An additional limitation is that we are not able to distinguish between comorbidity that had its onset during the pre-pregnancy period or during pregnancy versus the postpartum period. However, knowing the prevalence of comorbidity post-childbirth is still of value for birthing parents, their families, and their healthcare providers, given it can indicate their treatment needs and affect a parent's ability to care for their infant. A final limitation is that our sample was seeking treatment which could have increased comorbidity prevalence rates.

Given these findings, there may be value in researching transdiagnostic interventions as potential treatments for a significant proportion of those who are seeking treatment for PPD. Transdiagnostic treatments are those that focus on treating 2 disorders simultaneously.^58^ While they have gained attention in general population samples^59,60^ for their practicality, potential cost-effectiveness, and scalability, far fewer trials have been conducted in the perinatal period. Policymaking that treats postpartum comorbid depression and anxiety as the norm rather than the exception could also be important to ensure that services and guidelines are adequately structured to serve birthing parents with single or co-occurring diagnoses.

For treating clinicians, this level of comorbidity likely shapes how a large number of birthing parents present, as well as what the process and therapeutic targets will be. Treatment needs to be premised on a high potential for a blended clinical picture marked by persistent threat sensitivity and difficulty regulating worry in the context of profound role transition. Treating comorbid PPD and PPA is important not only from a public healthcare cost perspective but also to help families give their infants the strong start to life that they all wish to provide.

## Supplemental Material

sj-docx-1-cpa-10.1177_07067437261462698 - Supplemental material for Prevalence of Comorbid Postpartum Depression and Anxiety in Birthing Parents Seeking Treatment for Postpartum Depression Supplemental material, sj-docx-1-cpa-10.1177_07067437261462698 for Prevalence of Comorbid Postpartum Depression and Anxiety in Birthing Parents Seeking Treatment for Postpartum Depression by Karley V. George, BSc, Calan D. Savoy, MSc, Haley Layton, MPH, Peter J. Bieling, PhD, CPsych and Ryan J. Van Lieshout, MD, PHD in The Canadian Journal of Psychiatry
