# Supplementary material for: Prevalence of Comorbid Postpartum Depression and Anxiety in Birthing Parents Seeking Treatment for Postpartum Depression: Prévalence de la présence comorbide de la dépression et de l’anxiété post-partum chez les parents ayant accouché qui ont consulté en raison de la dépression post-partum
Source: Can J Psychiatry. 2026 Jul 1:07067437261462698. Online ahead of print. doi: 10.1177/07067437261462698 (PMC13323044; doi:10.1177/07067437261462698)
Supplement: sj-docx-1-cpa-10.1177_07067437261462698 - Supplemental material for Prevalence of Comorbid Postpartum Depression and Anxiety in Birthing Parents Seeking Treatment for Postpartum Depression [file sj-docx-1-cpa-10.1177_07067437261462698.docx]

**Supplementary Data**

| **Table S1.** Prevalence of Generalized Anxiety by Severity Level on the GAD-7 in Postpartum Participants with an EPDS ≥ 10 | | | | | | | | |
| --- | --- | --- | --- | --- | --- | --- | --- | --- |
| **Study** | **% low**  **(score 0-4)** | | **% mild**  **(score 5-9)** | | **% moderate  (score 10-14)** | | **% severe  (score >15)** | |
| Amani et al., 2021 | 8.5 | | 17.0 | | 36.2 | | 38.3 | |
| Van Lieshout et al., 2021 | 5.5 | | 27.4 | | 32.2 | | 34.9 | |
| Van Lieshout et al., 2023 | 8.7 | | 28.6 | | 33.3 | | 29.4 | |
| Babiy et al., 2024 | 3.7 | | 28.6 | | 29.8 | | 38.0 | |
| Merza et al., 2024 | 7.4 | | 24.1 | | 30.9 | | 37.7 | |
| Layton et al., 2025 | 6.1 | | 24.6 | | 31.6 | | 37.7 | |
| Mansoor et al. (manuscript) | 6.5 | | 32.8 | | 26.9 | | 33.9 | |
| **Pooled Prevalence** | **6.4** | | **27.9** | | **31.3** | | **35.0** | |
| 95% CI | 4.97-8.16 | | 25.74-30.08 | | 29.11-33.58 | | 32.08-37.97 | |
| Heterogeneity | I^2^ = 31.6%, p = 0.1865 | | I^2^ = 13.4%, p = 0.3279 | | I^2^ =0%, p = 0.7514 | | I^2^ = 23.7%, p = 0.2483 | |
|  | | | | | | | |  |
| Abbreviations: GAD-7 = Generalized Anxiety Disorder 7-item scale, EPDS = Edinburgh Postnatal Depression Scale, heterogeneity (I^2^) values of 25, 50 and 75 were considered low, moderate, and high, respectively, P-value < 0.05 | | | | | | | |  |
|  | | | | | | | |  |
|  | |  | |  | |  | |  |

|  | | | | |  |  |  |
| --- | --- | --- | --- | --- | --- | --- | --- |
|  |  |  |  | |  |  |  |
| **Table S2.** Cronbach’s Alphas for Included Studies | | | |  | |  |  |
| **Study** | | | | **EPDS Cronbach's α** | | **GAD-7 Cronbach's α** | **PSWQ Cronbach's α** |
| Amani et al. 2021 | | | | 0.74 | | 0.80 | n/a |
| Van Lieshout et al. 2021 | | | | 0.79 | | 0.85 | n/a |
| Van Lieshout et al. 2022 | | | | 0.78 | | n/a | 0.86 |
| Van Lieshout et al. 2023 | | | | 0.83 | | 0.85 | n/a |
| Huh et al. 2023 | | | | 0.83 | | n/a | 0.90 |
| Babiy et al. 2024 | | | | 0.83 | | 0.86 | n/a |
| Merza et al. 2024 | | | | 0.82 | | 0.87 | n/a |
| Layton et al. 2025 | | | | 0.80 | | 0.86 | n/a |
| Mansoor et al. (manuscript) | | | | 0.75 | | 0.85 | n/a |
| **Overall** | | | | **0.81** | | **0.85** | **0.88** |
|  | | | |  | |  |  |
| Abbreviations: α = alpha, EPDS = Edinburgh Postnatal Depression Scale, GAD-7 = Generalized Anxiety Disorder 7-item scale, PSWQ = Penn State Worry Questionnaire, n/a = measurement not used in the study | | | | | | | |
